# Detection of white head symptoms of panicle blast caused by *Pyricularia oryzae* using cut-flower dye

**DOI:** 10.1186/s13007-019-0548-z

**Published:** 2019-12-26

**Authors:** Keiko Hayashi, Tomofumi Yoshida, Yuriko Hayano-Saito

**Affiliations:** 1 NARO Central Region Agricultural Research Center, Kannondai, Tsukuba, Ibaraki 305-8666 Japan; 2 Mountainous Region Agricultural Institute, Aichi Agricultural Research Center, Inabu, Toyota, Aichi 441-2513 Japan

**Keywords:** Disease, Rice blast, Panicle blast, Leaf blast, Imaging

## Abstract

**Background:**

Breeding of rice with panicle resistance to rice blast disease caused by *Pyricularia oryzae* is a challenge towards sustainable rice production. Methods for accurate estimation of disease severity can support breeding. White head symptoms are a commonly used index of panicle blast in the field. As the development mechanism of this symptom remains unclear, we used cut-flower dye (CFD) solution to visualize the infected panicle tissues.

**Results:**

CFD delineated the edge of white head symptoms in rice panicles artificially infected with *P. oryza*e. Hyphae within the tissues were confirmed through staining with a fluorescent wheat germ agglutinin conjugate. Hyphal density was obviously diminished at the dye edge. Growing hyphae preferred to move along the vascular bundles; infected tissues lost the ability to transport water, leading to white head formation. By marking the edge of the white heads, this simple dyeing technique precisely reveals the extent of infection. Further, digital imaging allowed dried samples to be stored and reassessed later.

**Conclusions:**

The CFD detection technique served as a powerful tool for estimating disease severity by color, as it clearly revealed lesions in both the panicles and leaves. Combined with reliable methods for artificial inoculation and observation of infecting hyphae, this technique will advance the research and breeding of panicle blast-resistant rice.

## Background

Precise evaluation of disease severity can facilitate studies on resistance mechanisms and breeding for resistance. Understanding of the properties of a target disease is indispensable for accurate evaluation of disease severity.

Rice blast disease, which is caused by the fungus *Pyricularia oryzae*, inflicts serious damage to rice leaves (leaf blast) and panicles (panicle blast). Panicle blast is directly linked to rice production and quality; thus, resistance to this disease is important. However, genetic resources for panicle resistance are much more limited than those for leaf resistance because of the difficulties in field assay and the lack of a reliable artificial inoculation method.

The severity of leaf blast is generally estimated by measuring the damaged area and assessing the shape, size, color, and number of lesions on the leaves. Lesion properties have been studied in both susceptible and resistant cultivars [1]. Typical lesions in susceptible cultivars are elliptical with more or less pointed ends, a gray or whitish center, and a brown or reddish-brown margin [1]. Lesions have three zones (from the edge to the center): venenate (meaning poisoned, derived from the word “venin”), necrotic, and disintegrated zones [2]. The venenate zone is yellow and poisonous, as it contains toxins secreted by the fungus. The necrotic zone is brown owing to degeneration of cell components. The disintegrated zone is completely collapsed. Browning indicates a hypersensitive reaction in resistant cultivars and the presence of factors that impede lesion development [1]. Browning is caused by serotonin accumulation, which attenuates oxidative stress at the lesion [3]. The use of fungus modified with a fluorescent protein tag, which can be imaged directly as it invades leaf tissues, can help elucidate the mechanism of leaf blast resistance [4–6].

The properties of panicle blast lesions are less well known than those of leaf blast because the information on how hyphae invade panicles is limited owing to the following technical problems. Panicle tissue is hard to slice for observation under a microscope, and there is no reliable artificial infection method using fluorescence-tagged pathogen transformants. Moreover, all parts of the panicle—spikelet, glume, branches, rachis, and neck—are susceptible to blast disease; in particular, damage to the neck (neck blast) leads to complete loss of the panicle [1, 7–10]. Infection of the branches, rachides, and neck causes white head symptoms owing to discoloration of the tissues distal of the infection site [2, 9]. Unlike in leaf blast, there are few typical lesions in panicles. Investigation of fungus dynamics in the panicles would clarify its mechanism of infection, and provide useful information for breeding rice resistant to panicle blast.

The severity of panicle blast in the field is generally judged by visual assessment of the area of white head symptoms [11–13], which provides a useful information on how the fungus invades the panicles. It is difficult to analyze symptoms in a large number of panicle samples, as rice tissues wither quickly and lose color once picked; therefore, we used a cut-flower dye (CFD) solution to delineate damaged tissues. CFDs are commonly used to change the color of cut flowers: white cut flowers can be dyed any color through immersion in a CFD solution. As the CFD is transported through the xylem to all tissues, we thought that dyeing would allow visualization of damaged tissues, which cannot transport water.

We revealed that CFD delineated the edge of white head symptoms induced by artificial infection. The accuracy of delineation was confirmed through detection of hyphae stained with a fluorescent dye: hyphal density was obviously diminished at the dye edge, as growing fungus collapsed or blocked the vascular bundles, blocking the advance of the CFD, which thus built up at the edge of the lesions. The CFD enabled us to accurately detect symptoms in samples infected naturally in the field. The technique was easily adapted to digital image analysis of both fresh and dry tissues, allowing severity assessment of panicle blast at any time.

## Results

Hyphal staining with the fluorescent wheat germ agglutinin (WGA) conjugate was confirmed in fungus grown on agar. Hyphae were observed growing inside the medium (Additional file 1: Fig. S1a). However, spore fluorescence was undetectable (Additional file 1: Fig. S1b).

**Fig. 1:**
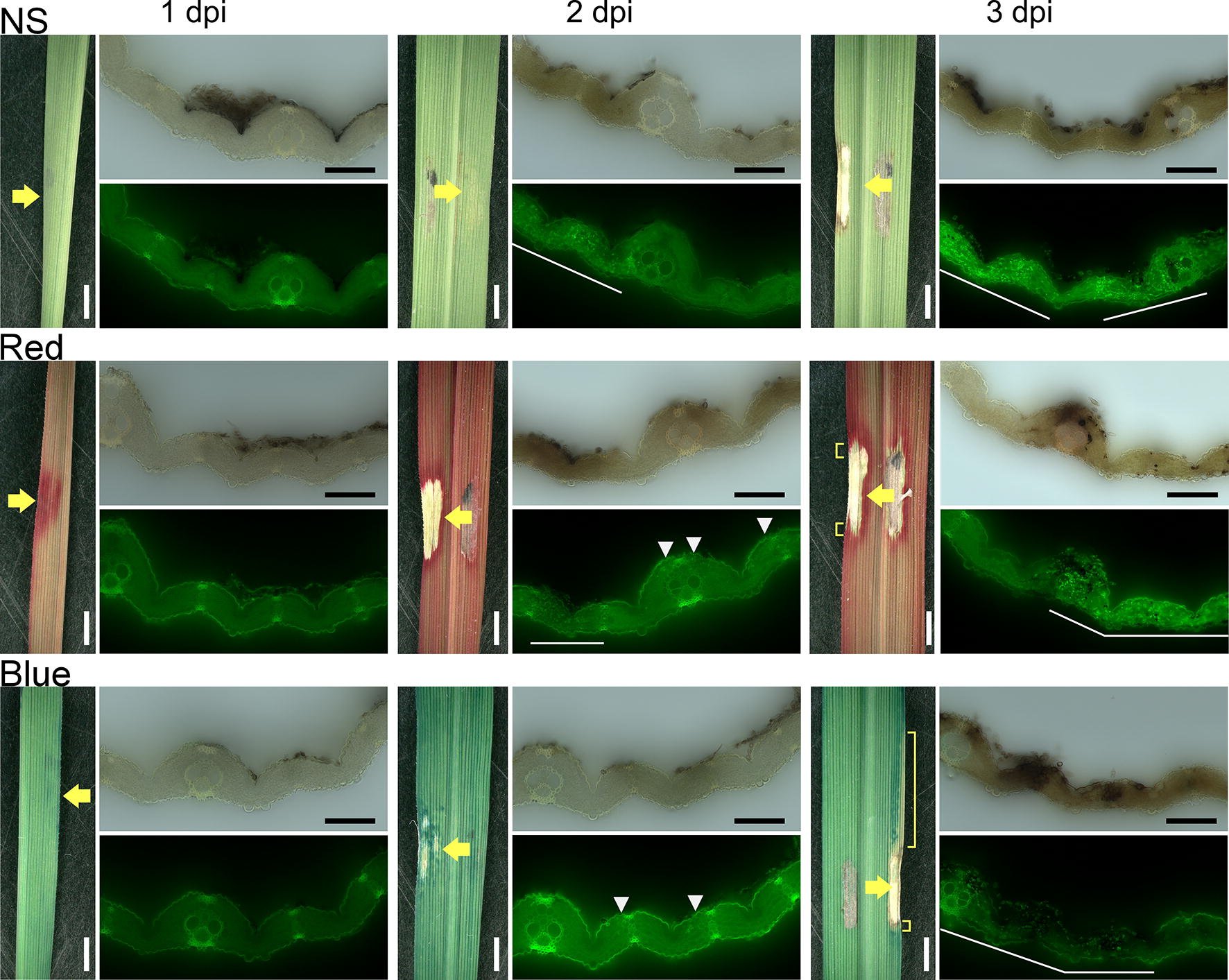
Leaves damaged by rice blast fungus were dyed. Leaves were either treated with water (NS, no staining) or dyed with red or blue cut-flower dye at 1 to 3 days post-inoculation (DPI). The lower (abaxial) side of the leaf blades is shown. Yellow arrows indicate target lesions. Yellow brackets mark areas that retain the original leaf tissue colors. Upper images in each pair show light microscopy results; lower images show fluorescence microscopy results. Spores and appressoria appeared on the upper epidermis. White arrowheads and white lines indicate hyphae detected with fluorescent wheat germ agglutinin conjugate. White bars, 2000 µm; black bars, 100 µm

Before evaluating the effect of CFD solution on panicle blast, we stained rice leaves with red, blue, and green CFDs to elucidate the relation between leaf lesions and dye patterns. We observed the dye patterns of CFDs at leaf blast lesions induced by spot-inoculation. Lesions were observed from the lower side of the leaf blade to avoid the spore clump at the inoculation site. The location of the cells directly under the inoculation site was determined by the position of appressoria. At 1 day post-inoculation (DPI), the inoculation site in undyed samples showed no critical change, but that in some dyed samples showed coloration (Fig. 1; Additional file 2: Fig. S2a). There was no severe cell destruction, and no hyphae were observed in the cells directly under the inoculation site. At 2 DPI, the undyed inoculation sites had yellowed slightly, and the dyed sites were clearly visible. Growing hyphae were visible in the cells between vascular bundles. At 3 DPI, lesions, including a necrotic zone, were clearly evident and surrounded by strong coloration. Tissues, including vascular bundles, had collapsed and were filled with hyphae, forming a disintegrated zone [2]. The dyed margin was larger than the undyed margin of the necrotic zone.

**Fig. 2:**
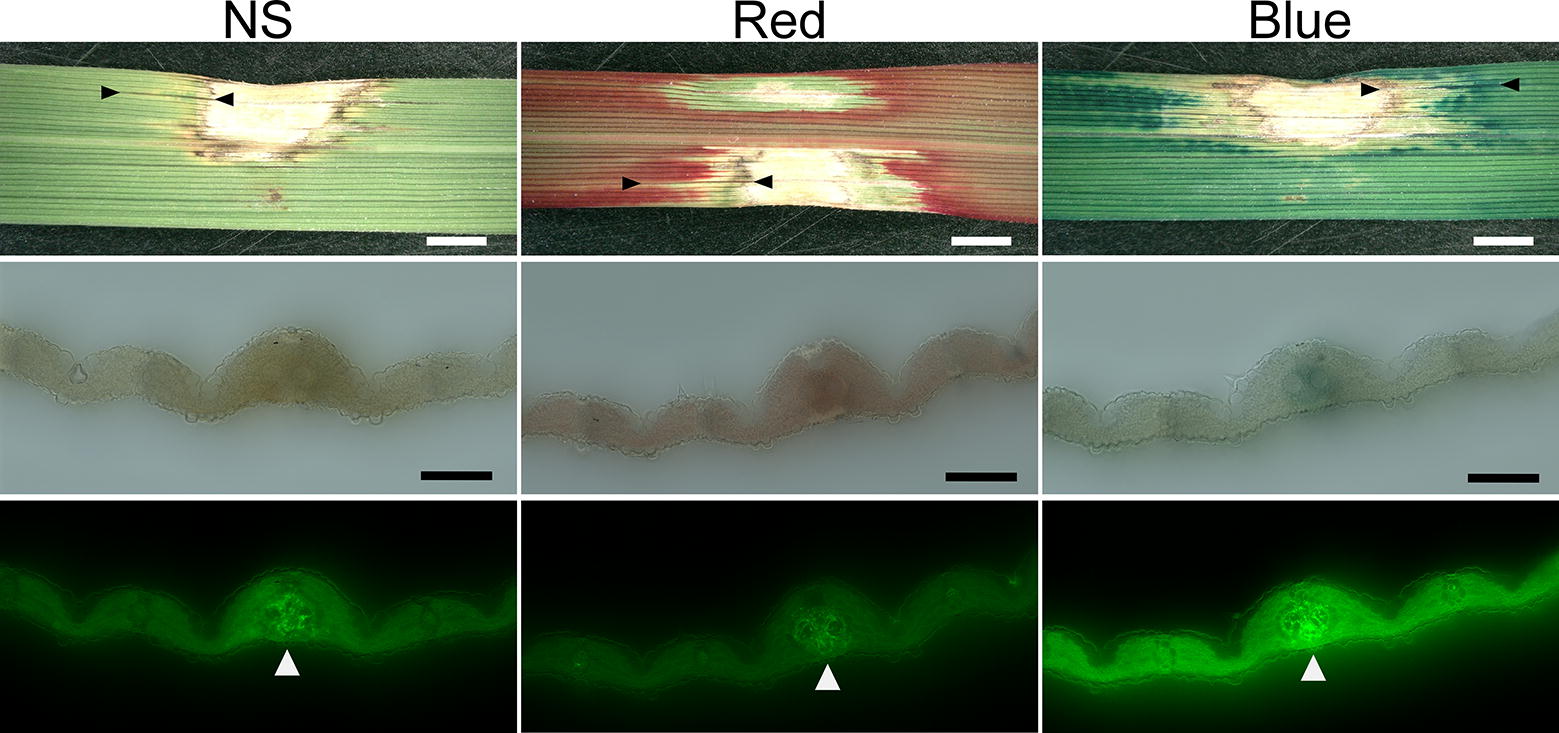
Lesions with lines of necrosis appeared from 5 DPI. Leaves were treated with water (NS, no staining) or dyed with red or blue cut-flower dye. Upper row: the lower (abaxial) side of the leaf blades is shown. The region between the two black arrowheads in each upper image is an example of a necrosis line. Middle row: cross-sections observed by light microscopy. Lower row: cross-sections observed by fluorescence microscopy. White arrowheads indicate hyphae detected with fluorescent wheat germ agglutinin conjugate. White bars, 2000 µm; black bars, 100 µm

A necrosis line is a characteristic used to distinguish rice blast from other fungal lesions. Lines appeared from 5 DPI, and were made obvious by dyeing (Fig. 2; Additional file 2: Fig. S2b; Additional file 3: Fig. S3a). In 41 lines of necrosis derived from independent lesions, hyphae were detected in cells close to vascular bundles.

**Fig. 3:**
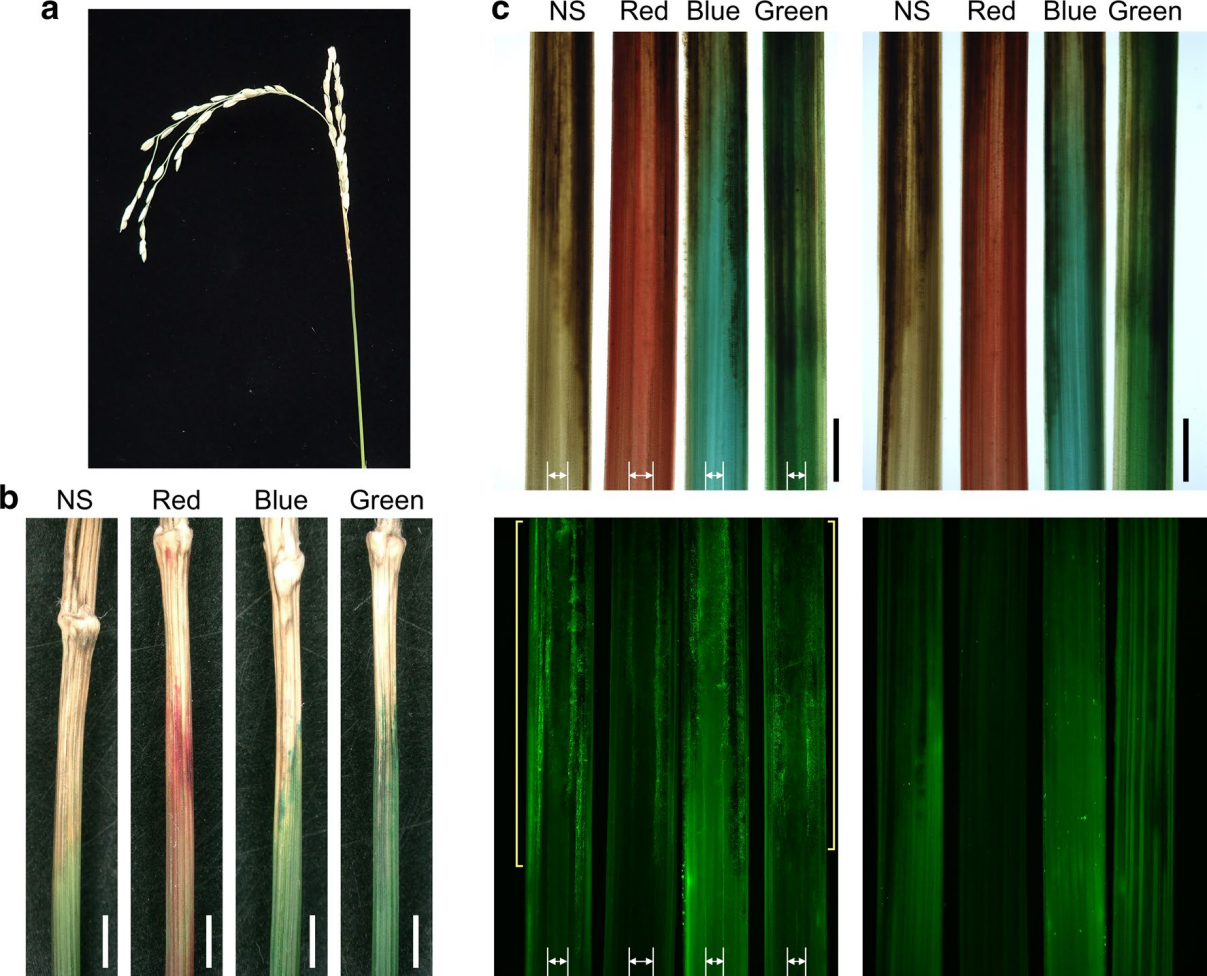
Panicles damaged by rice blast fungus were dyed. **a** Panicle with neck damage. **b** Necks were treated with water (NS, no staining) or dyed with red, blue, or green cut-flower dye. **c** Necks were cut longitudinally through the center. Upper panels: light microscopy images; lower panels: fluorescence microscopy images. Left: the inside of the neck; right, the surface of the neck. Long yellow brackets indicate hyphae detected with fluorescent wheat germ agglutinin conjugate. Double-headed arrows indicate internal lacunae. White bars, 2000 µm; black bars, 100 µm

In the plastic greenhouse, white head symptoms appeared on the panicle branches and neck at 2 and 3 weeks after spraying, respectively. The damaged areas did not take up dye, but the undamaged tissues bordering them were strongly dyed (Fig. 3a, b). Panicle necks cut longitudinally through the center were stained with WGA conjugate for fluorescence microscopy (Fig. 3c). The damaged regions remained brown and were filled with hyphae, which diminished toward the undamaged tissue. Hyphae were present in the air lacunae of the necks. Few hyphae were observed on the surface of the heavily infected necks. Hyphae were detected in not only vascular bundles but also the intercellular spaces of the neck (Additional file 3: Fig. S3b).

**Fig. 4:**
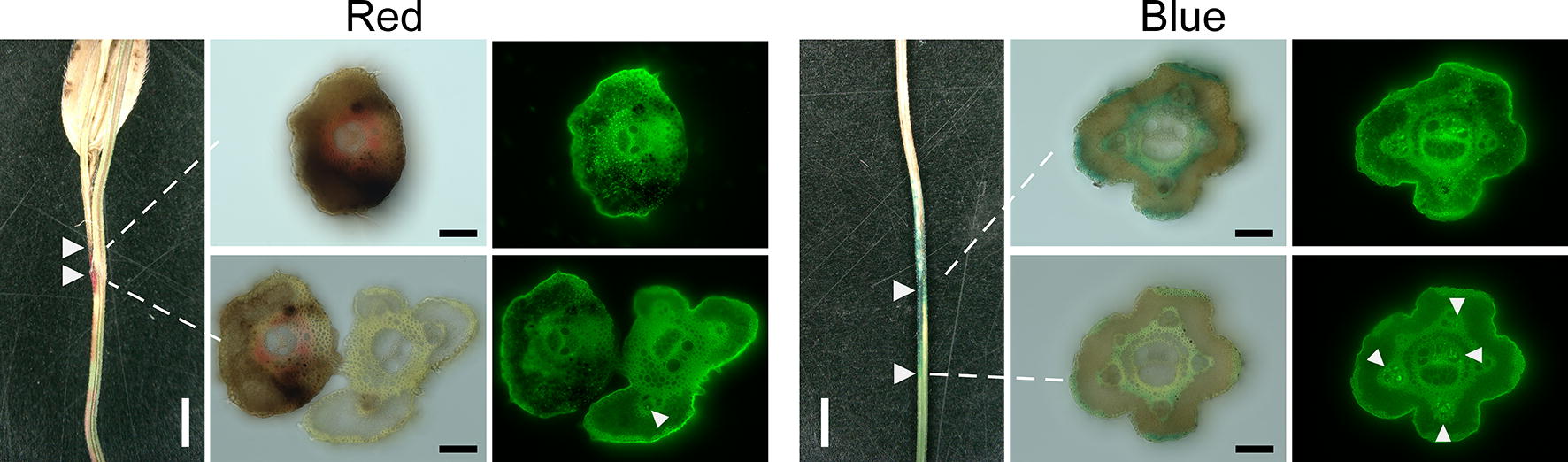
Panicle branches damaged by rice blast fungus were dyed with red or blue cut-flower dye. Left panels: the border of the dyed tissues was cut in cross-section (arrowheads); central panels: light microscopy images; right panels: fluorescence microscopy images (fluorescent wheat germ agglutinin conjugate). Upper panels: high hyphal density; lower panels: low hyphal density. White arrowheads in fluorescent images indicate hyphae detected in vascular bundles. White bars, 2000 µm; black bars, 100 µm

Dye and hyphal patterns in the panicle branches were similar to those in the necks (Fig. 4; Additional file 2: Fig. S2c). Hyphae were detected within the branches but not on the surface. Some hyphae were identified in dyed vascular bundles close to the edge of the dye (Fig. 4), but no hyphae were identified below the dye (Additional file 2: Fig. S2c). The results showed that CFDs delineated the edges of the lesions in the same manner in all parts of the panicles.

**Fig. 5:**
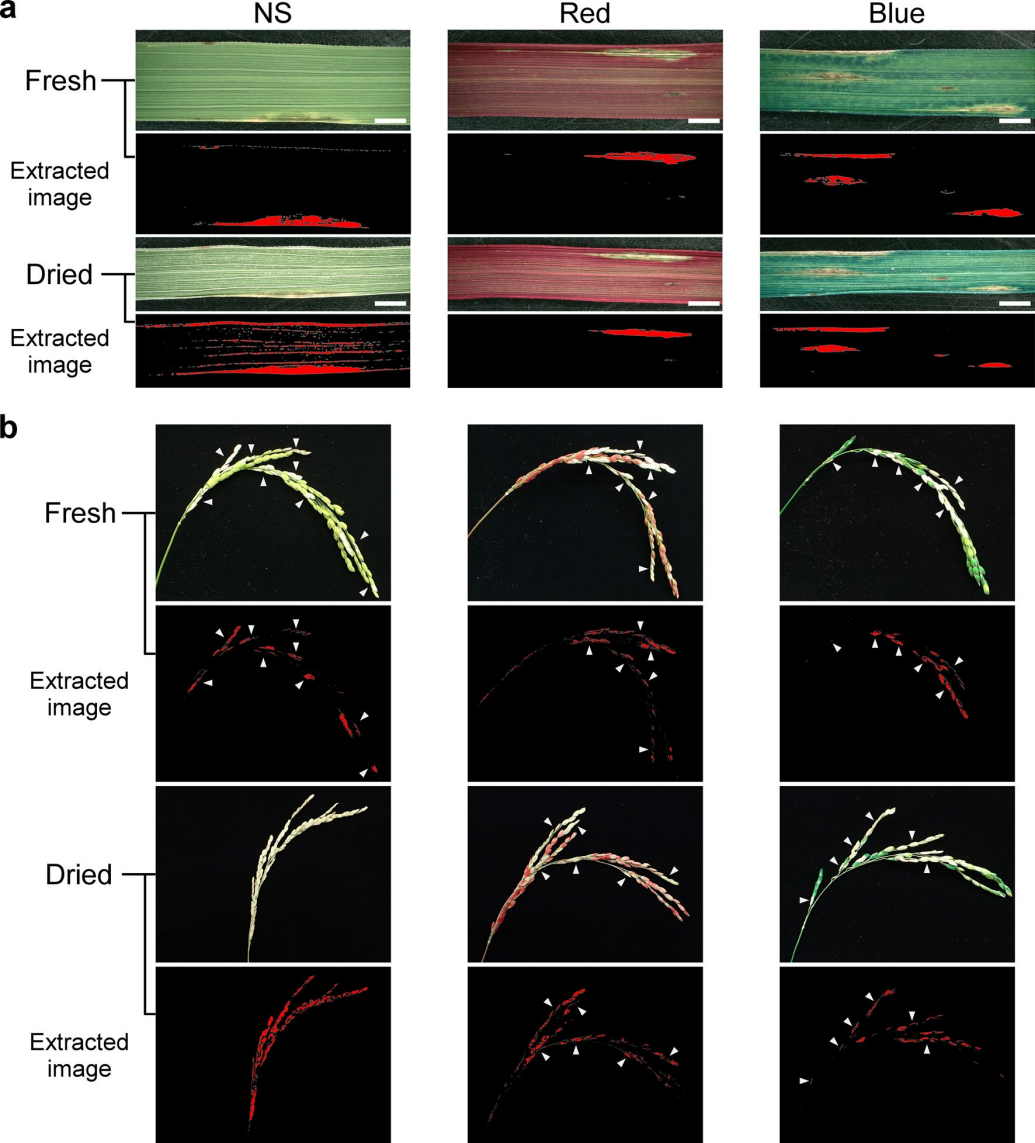
Digital imaging of **a** leaf and **b** panicle blast. Samples were treated with water (NS, no staining) or dyed with red or blue cut-flower dye. The red-on-black images, extracted using the Keyence microscopy software (“Extracted image”), correspond to lesions in fresh (upper) and dried (lower) samples. Arrowheads indicate damaged parts of the panicles. White bars, 2000 µm

Spray-inoculated leaves developed both necrotic lesions, in which the fungus was expanding, and brown lesions, in which the fungus failed to expand. Digital color extraction clearly detected both types of lesions in stained fresh and dried leaves as well as in unstained fresh leaves, but less accurately in unstained dried leaves (Fig. 5a; Additional file 2: Fig. S2d). In fresh panicles, the accuracy of color extraction did not differ among panicles treated with water and with red, blue, or green CFD (Fig. 5b; Additional file 2: Fig. S2e). In dried panicles treated with water, the total panicles were extracted because it was difficult for the software to distinguish between damaged and undamaged tissues owing to discoloration of the panicles. Nevertheless, this method accurately extracted the damaged areas of dyed panicles.

To evaluate the effectiveness of CFD in detecting rice susceptibility to panicle blast under artificial conditions, we dyed infected panicles collected in a paddy field and used a fluorescent WGA conjugate to confirm the detection (Table 1). Hyphae were identified at the dyed edge of the branches, rachides, and necks, as well as in and around vascular bundles in many cross-sections. The patterns of dye and hyphal fluorescence were the same as in artificially infected samples.

**Table 1:** Detection of hyphal fluorescence in infected panicles collected in a paddy field

Panicle position	No. of samples	Strongly detected^a^	Weakly detected^a^	Not detected^a^	No. of detections in and around vascular bundles^b^
Branch	31	30	1	0	29
Rachis	33	33	0	0	26
Neck	17	16	1	0	16

^a^Strongly detected, strong fluorescence of hyphae; weakly detected, weak fluorescence; not detected, no fluorescence

^b^No. of samples when hyphae were identified in at least one cross-section

## Discussion

White head symptoms indicate severe damage to the panicles. The symptoms might be caused by blockage of water transport due to collapsed vascular bundles [2, 8, 9]; thus, using CFDs to stain the panicles, we confirmed the blockage of water transport at the edge of lesions.

The disintegrated zone of the lesions was undyed owing to fungus-induced destruction of leaf tissues and blockage of vascular bundles (Fig. 1; Additional file 2: Fig. S2a). In the leaves, the dyed margin was larger than the undyed margin (Fig. 1; Additional file 2: Fig. S2a); therefore, the damaged region might be larger than it appeared and the venenate zone may have lost its ability to transport water even though the cells appeared intact. The cells surrounding the necrosis line were undyed (Fig. 2; Additional file 2: Fig. S2b; Additional file 3: Fig. S3a). These results suggested that fungal invasion in vascular bundles blocked water transport. The lesions expanded as hyphae grew along the vascular bundles, and the hyphae blocked the movement of the CFD, which thus accumulated at the edge of the lesions.

CFD delineated white head symptoms the same way in the panicles as in the leaves. In distal symptomatic parts, hyphae were detected mainly in vascular bundles and the surrounding cells. In the neck, hyphae were also present in the air lacunae (Fig. 3c). Despite the mass of fungus within tissues, few hyphae were observed on the surface at the edge of the symptomatic parts. As hyphae in leaves were present mainly in vascular bundles within necrosis lines, hyphae in the panicles might also grow along vascular bundles. Moreover, rice blast fungus did not completely destroy vascular bundles in infected panicle tissues around the edge of the symptomatic areas (Fig. 4; Additional file 2: Fig. S2c; Additional file 3: Fig. S3b), consistent with previous reports [7–9]. In plate culture, the fungus grew along the sclerenchymatous fibers in cut neck tissue [14]. These results suggested that rice blast fungus preferred to move long distances through tissues inside the panicle, such as vascular bundles and air lacunae, and the site of invasion may be different from the edge of the white head symptoms.

Direct evaluation of the proportion of diseased grains is the best way to estimate the severity of panicle blast [15]. In the field, it is based on a visual assessment [16]; the area of damage is used as an index of the proportion [11–13]. This assay is useful for breeding, but an objective criterion is needed to elucidate the mechanisms of resistance. The area of damage in the panicles dyed with CFDs can be identified and scored accurately in both artificially and naturally infected samples. Dyeing thus served as a new tool for analyzing rice resistance to panicle blast by differentiating the severity of damage among cultivars and lines. The detection of hyphae by staining with a fluorescent dye will further contribute to the analysis of panicle blast.

Fluorescent protein (e.g. GFP)-tagged pathogens are routinely used to observe plant–microbe interactions in leaf and root tissues [4, 5, 17, 18]. However, panicle blast must be detected in field samples. Hyphae marked with a fluorescent WGA conjugate in cleared tissues were clearly visible under a fluorescence microscope in both leaf and panicle samples. Because rice blast fungus is not a specific target of WGA–fluorescein conjugate, other fungi could be misidentified as rice blast fungus in the field [19, 20]. Thus, analysis of artificially infected samples by WGA conjugate could allow confirmation. The fungal dynamics following artificial infection appeared to parallel those following natural infection. Therefore, artificial inoculation with fluorescently tagged pathogens would accelerate the studies of plant–fungus interactions.

Strict evaluation of disease severity in a large number of samples is difficult. Digital imaging allows color to be recorded, but lesions can have various colors, and images need elaborate digital processing to delineate lesions [21]. There were no significant differences in dyeing patterns or accuracy of digital segmentation among the three CFDs in either leaf or panicle samples. Red CFD allowed easy assessment of the degree of staining, but it masked hyphal fluorescence. Thus, color did not affect the quality of detection, but selection of color should depend on the purpose of the sample detection.

CFD could be a powerful tool for detecting damage by rice blast. It offered a simple, prompt, and accurate method to delineate lesions on the leaves and panicles without the need for special skills. A popular CFD, Flower Palette (Torii Co., Ltd., Aichi, Japan), was able to reveal leaf and panicle blast even after 6 months of storage in the dark (data not shown). Thus, various CFDs can be used to detect rice blast. The availability of various colors allows CFDs to be used flexibly depending on the stage of development and the color of target plants.

These methods could allow evaluation of complex targets, such as the effects of gene pyramiding and field resistance, in other diseases causing structurally similar lesions as those of rice blast, as well as examination of physiological responses to drying and water stress. These methods could help visualize a wide range of plant properties, basic mechanisms of host–pathogen interaction, plant physiology, and hydrodynamics.

## Conclusion

We have shown a simple method using CFDs to clearly visualize the proportion of white head symptoms, which is a generally used index of panicle blast in the field. We confirmed the mechanism of this method, as well as its accuracy and reliability. Stained samples, including not only panicle samples but also leaf samples, were ideal for digital imaging analysis; moreover, the dried samples can be stored for a long period. In combination with artificial infection and fluorescent staining of hyphae, the CFD detection technique can advance our understanding of resistance to panicle blast and other plant diseases that result in structurally similar lesions, as well as the breeding of blast-resistant rice.

## Materials and methods

### Plant growth conditions and inoculation with leaf blast

We inoculated the leaves of the susceptible cultivar ‘Nipponbare’ through both spot-inoculation and spray inoculation at the 6½-leaf stage in a glasshouse.

The leaves were spot-inoculated as described previously [3] with modifications. An extending leaf was marked on the upper side with a black marker pen to indicate the inoculation site. The site was wiped softly with a cotton soaked with 0.05% (v/v) Tween-20 to remove leaf wax, and a drop of spore suspension (*P. oryzae*, Ao-9-06-2, race 337.1) was placed on this site. The date was defined as 0 DPI. After 24 h of incubation at 25 °C in the dark under high humidity, the plants were maintained in a glasshouse at 25 °C until staining with CFDs.

The leaves were also spray-inoculated as described previously [22], using the same race. The inoculated plants were held in a glasshouse at 25 °C until staining with CFDs.

### Plant growth conditions and inoculation with panicle blast

We artificially inoculated the panicles of the susceptible cultivars ‘Nipponbare’ and ‘Koshihikari’ with blast fungus and stained them as described below. Six plants per pot (113-mm wide, 140-mm tall; Fujiwara Co., Tokyo, Japan) were grown, retaining only the main tiller, under natural conditions in a glasshouse. After heading, the plants were transferred into a hand-made plastic greenhouse (75-cm wide, 90-cm long, 140-cm tall; Additional file 4: Fig. S4) covered with clear polyethylene film (0.03-mm thick; FC-50, Okura Industry Ltd. Co., Kagawa, Japan) within the glasshouse at 25 °C for inoculation, as infection at heading leads to severe damage to the panicles [23, 24]. To reproduce conditions similar to those in the field, in which rice blast spores are dispersed and adhere to the host mainly at night and in the early morning under high humidity [25], we sprayed all panicles within the plastic greenhouse with 100 mL of water containing 1 × 10^5^ spores mL^−1^ once a day in the late afternoon (~ 16:00) for 4 days, and misted them for 3 min before sunrise (~ 05:00) to keep them wet in the morning (average humidity at night, 79.5%). The water did not contain surfactant; thus, the spores would adhere naturally. After 4 days, the plants were misted for an additional 3 min in the evening (~ 20:00) to raise the night-time humidity. The polyethylene film was lifted during daytime to reduce high-humidity stress on the panicles. Symptoms were assessed 2 weeks after spraying.

### Infection with panicle blast in experimental paddy field

Samples of panicle blast were collected in an experimental high disease-pressure paddy field at the Aichi Agricultural Research Center, Mountainous Region Agricultural Institute (35° 12.7′ N, 137° 30.4′ E). Fifteen ‘Koshihikari’ plants were transplanted by hand in 90-cm-long rows at 30 cm intervals. Diseased panicles were collected starting from 2 weeks after the heading date.

### Cut-flower dye staining

The infected leaf blades were cut at the base of the blade, and the cut ends were placed in a CFD solution (Flower Fantasy; Palace Chemical Co., Ltd., Yokohama, Japan) for 30 min to 3 h at 25 °C in the light under low humidity. The infected panicles were cut from 15 to 20 cm below the neck and then placed in CFD for 3 to 6 h under the same conditions as the leaves. The dyeing duration differed with the degree of leaf extension and the time after panicle heading. Dyeing was considered complete according to the degree of staining.

### Wheat germ agglutinin staining of hyphae

After the leaves and panicles showed uptake of the CFDs, they were for viewing using Tomei Tissue-Clearing Reagent for Plants (Tokyo Chemical Industry Co., Ltd.) according to the manufacturer’s instruction to maximize the detection of fluorescence intensity [26]. First, they were fixed overnight in acetic acid: ethanol (1:3), washed with 70% ethanol, then with 30% ethanol in phosphate-buffered saline (PBS) (Nippon Gene Co., Ltd.), and finally rinsed twice with PBS. The inoculation site was cut (~ 0.25- to 0.5-mm wide) in cross-section (leaf and panicle) or longitudinally through the center (panicle) using a stainless-steel razor blade. The sections were treated with 5 µg/mL Alexa Fluor 488–WGA conjugate (Invitrogen) for 1 h and then rinsed twice with PBS. The sections were then steeped in undiluted Tomei Tissue-Clearing Reagent for at least 1 day in the dark. The sections were observed under an all-in-one fluorescence microscope (BZ-X700; Keyence; GFP or TRITC filter was set).

Hyphae were also grown on potato dextrose agar with and without WGA and on oatmeal agar medium, as described previously [22], for verification of staining.

### Digital imaging

We took digital images of the dyed leaves of ‘Nipponbare’ and panicles of ‘Koshihikari’ with a digital camera in macro mode (Tough TG-5; Olympus) and processed them in a digital microscope software (VHX-5000; Keyence) to extract the regions corresponding to damaged tissues by hue (leaf, 22–59; panicle, 30–42), saturation (leaf, 0–90; panicle, 0–54), and brightness (leaf, 128–255; panicle, 135–255).

## Supplementary information

**Additional file 1: Fig. S1.** Detection of rice blast fungus grown in medium with Alexa Fluor 488–wheat germ agglutinin (WGA) conjugate. Left, light microscopy images; right, fluorescence microscopy images. **a** Hyphae grown on potato dextrose agar with (left) and without WGA (right) were sliced and observed. **b** Spores (white arrowheads) on oatmeal agar medium. Black bar, 500 µm; white bar, 50 µm.

**Additional file 2: Fig. S2.** Examples of leaf and panicle tissues dyed with green cut-flower dye. **a** to **e** correspond to Figs. 1, 2, 4, 5a, and 5b, respectively.

**Additional file 3: Fig. S3.** Hyphae detected in vascular bundles of the leaf and neck. **a** Cross-sections of necrosis lines in the leaves (left) were observed by fluorescence microscopy (right). White arrowheads indicate hyphae detected in large and small vascular bundles. **b** Cross-sections of the neck observed at high resolution under an all-in-one fluorescence microscope (BZ-X700; Keyence) with a sectioning module. Upper left: light microscopy image. Lower left: image observed through a GFP filter set. Upper right: image observed through the TRITC filter set. Lower right: overlaid images showing the margin. White arrowheads in the fluorescent images indicate hyphae detected in the intercellular spaces. Black bar, 100 µm.

**Additional file 4: Fig. S4.** A hand-made greenhouse covered with clear polyethylene film.

## Data Availability

The data supporting the conclusions of this article are included within the article and the additional file.

## Abbreviations

PBS: Phosphate-buffered saline
WGA: Wheat germ agglutinin
DPI: Days post-inoculation
GFP: Green fluorescent protein
