# Supplementary figures and images for: Detection of white head symptoms of panicle blast caused by Pyricularia oryzae using cut-flower dye
Source: Plant Methods. 2019 Dec 26;15:159. doi: 10.1186/s13007-019-0548-z (PMC6931245; doi:10.1186/s13007-019-0548-z)

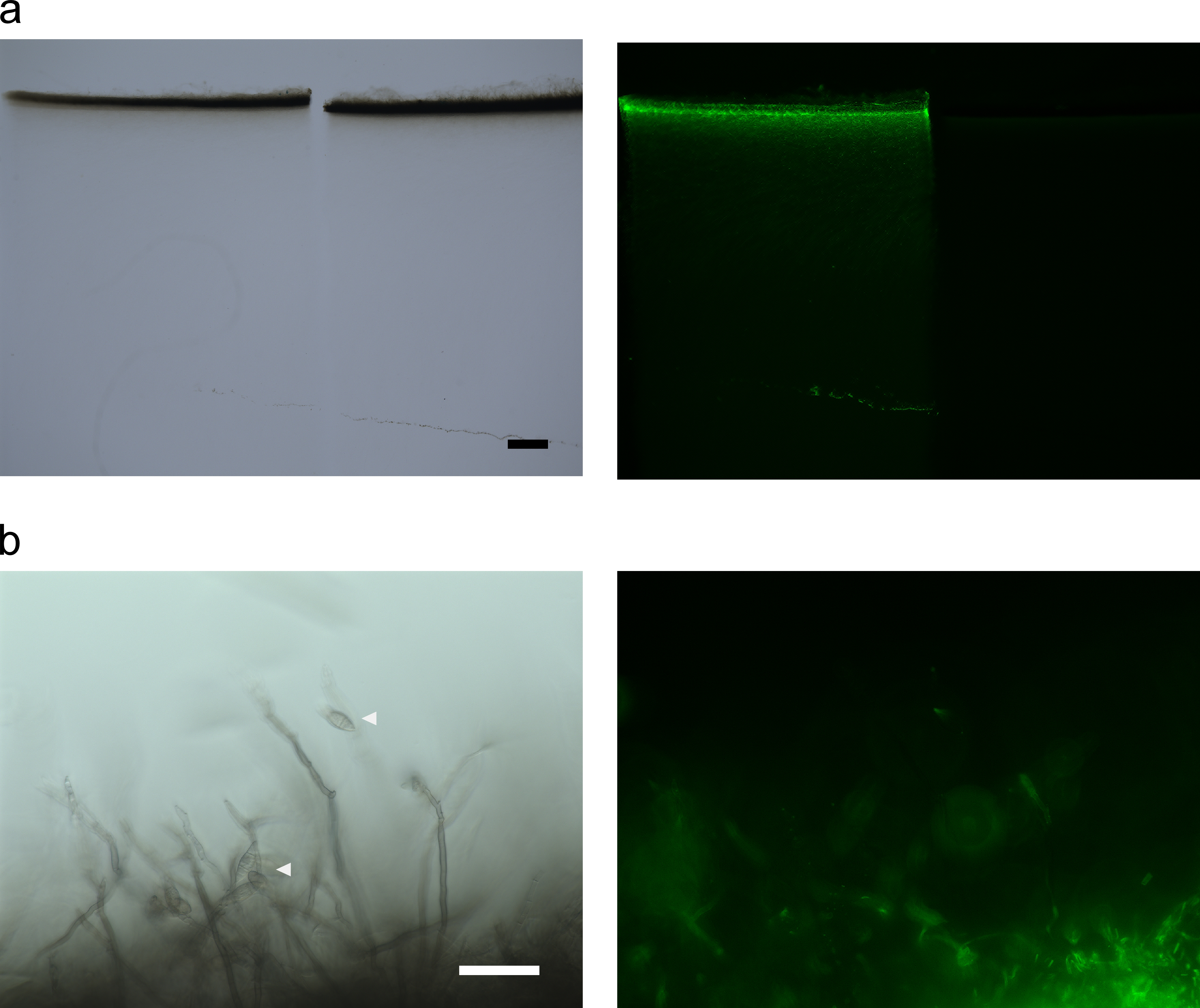

Supplement: Supplementary file 1 — Additional file 1: Fig. S1. Detection of rice blast fungus grown in medium with Alexa Fluor 488–wheat germ agglutinin (WGA) conjugate. Left, light microscopy images; right, fluorescence microscopy images. a Hyphae grown on potato dextrose agar with (left) and without WGA (right) were sliced and observed. b Spores (white arrowheads) on oatmeal agar medium. Black bar, 500 µm; white bar, 50 µm. [file 13007_2019_548_MOESM1_ESM.jpg]

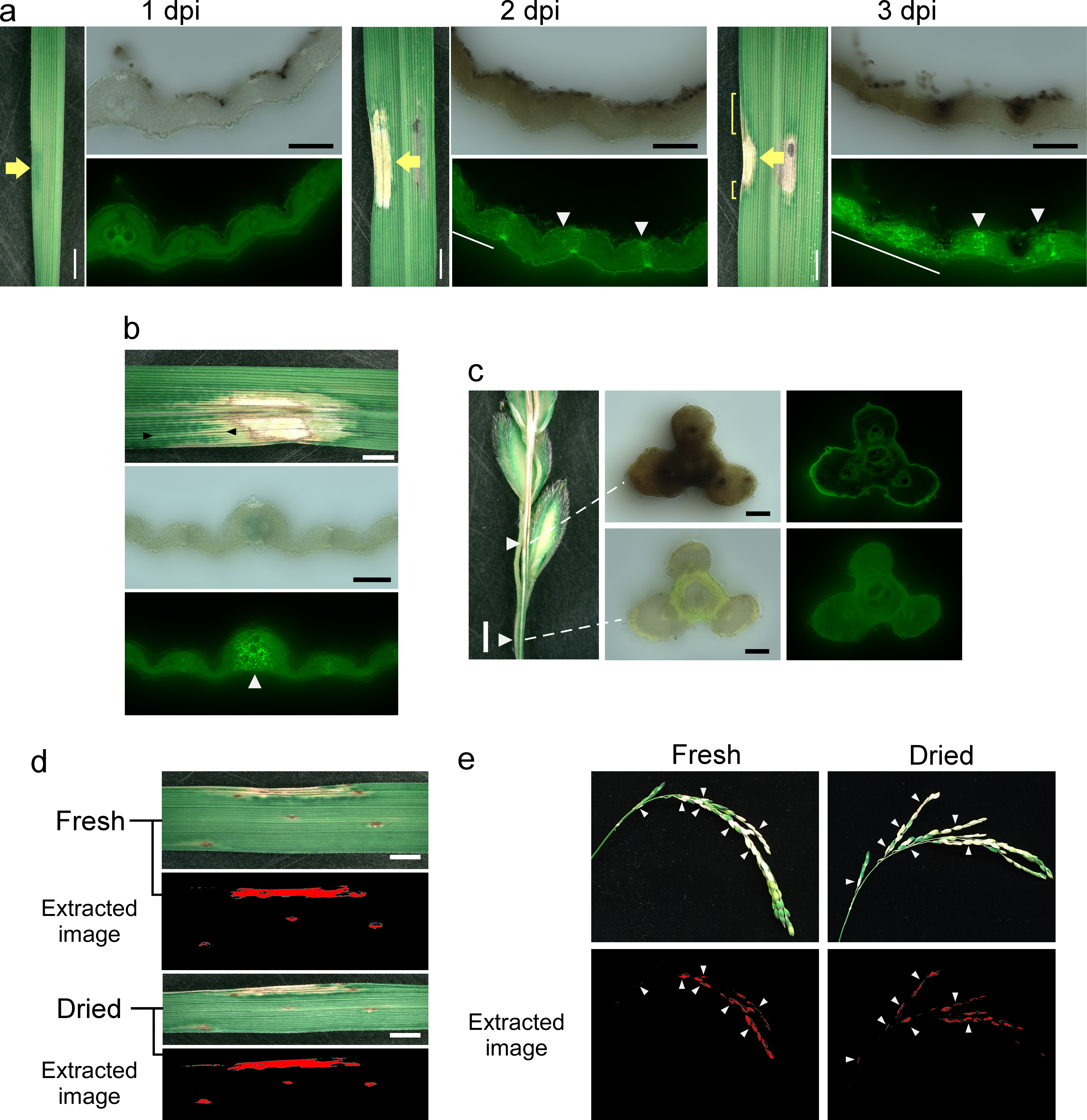

Supplement: Supplementary file 2 — Additional file 2: Fig. S2. Examples of leaf and panicle tissues dyed with green cut-flower dye. a to e correspond to Figs. 1, 2, 4, 5a, and 5b, respectively. [file 13007_2019_548_MOESM2_ESM.jpg]

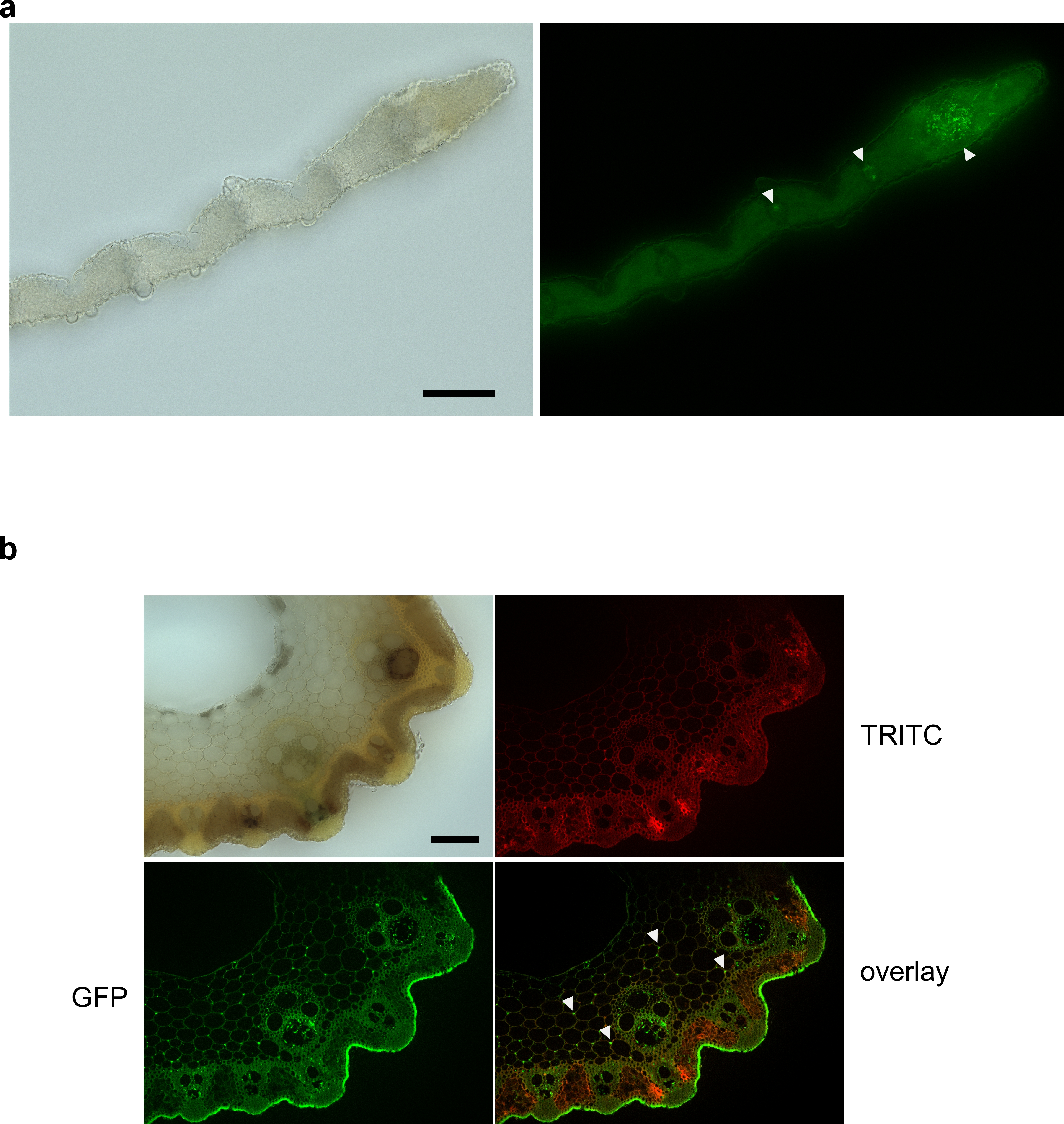

Supplement: Supplementary file 3 — Additional file 3: Fig. S3. Hyphae detected in vascular bundles of the leaf and neck. a Cross-sections of necrosis lines in the leaves (left) were observed by fluorescence microscopy (right). White arrowheads indicate hyphae detected in large and small vascular bundles. b Cross-sections of the neck observed at high resolution under an all-in-one fluorescence microscope (BZ-X700; Keyence) with a sectioning module. Upper left: light microscopy image. Lower left: image observed through a GFP filter set. Upper right: image observed through the TRITC filter set. Lower right: overlaid images showing the margin. White arrowheads in the fluorescent images indicate hyphae detected in the intercellular spaces. Black bar, 100 µm. [file 13007_2019_548_MOESM3_ESM.jpg]

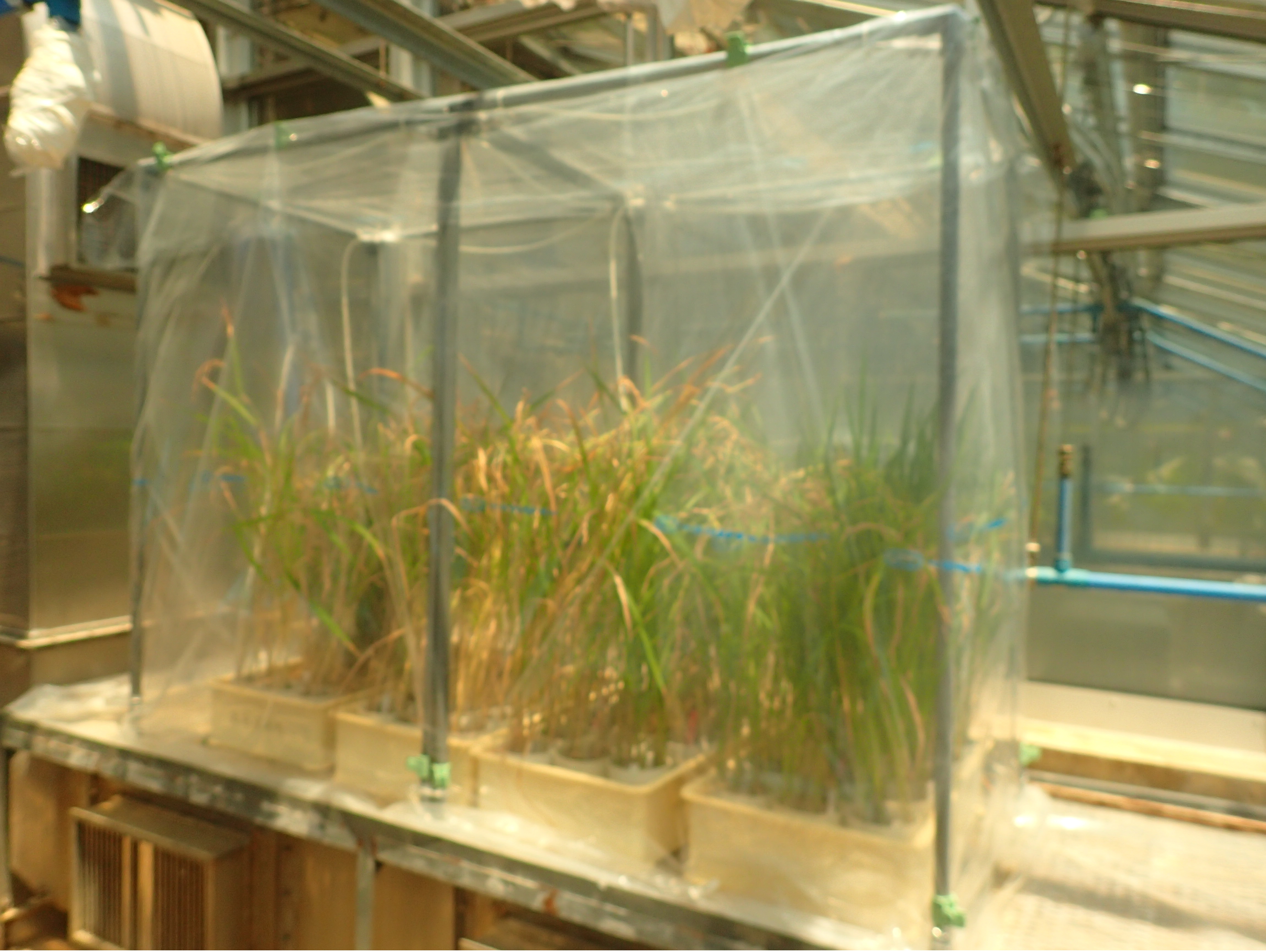

Supplement: Supplementary file 4 — Additional file 4: Fig. S4. A hand-made greenhouse covered with clear polyethylene film. [file 13007_2019_548_MOESM4_ESM.jpg]
